# Insights into Pancreatic Cancer Etiology from Pathway Analysis of Genome-Wide Association Study Data

**DOI:** 10.1371/journal.pone.0046887

**Published:** 2012-10-04

**Authors:** Peng Wei, Hongwei Tang, Donghui Li

**Affiliations:** 1 Division of Biostatistics and Human Genetics Center, School of Public Health, University of Texas Health Science Center, Houston, Texas, United States of America; 2 Department of Gastrointestinal Medical Oncology, The University of Texas MD Anderson Cancer Center, Houston, Texas, United States of America; Vanderbilt University Medical Center, United States of America

## Abstract

**Background:**

Pancreatic cancer is the fourth leading cause of cancer death in the U.S. and the etiology of this highly lethal disease has not been well defined. To identify genetic susceptibility factors for pancreatic cancer, we conducted pathway analysis of genome-wide association study (GWAS) data in 3,141 pancreatic cancer patients and 3,367 controls with European ancestry.

**Methods:**

Using the gene set ridge regression in association studies (GRASS) method, we analyzed 197 pathways identified from the Kyoto Encyclopedia of Genes and Genomes database. We used the logistic kernel machine (LKM) test to identify major contributing genes to each pathway. We conducted functional enrichment analysis of the most significant genes (*P*<0.01) using the Database for Annotation, Visualization, and Integrated Discovery (DAVID).

**Results:**

Two pathways were significantly associated with risk of pancreatic cancer after adjusting for multiple comparisons (*P*<0.00025) and in replication testing: neuroactive ligand-receptor interaction, (*Ps*<0.00002), and the olfactory transduction pathway (*P* = 0.0001). LKM test identified four genes that were significantly associated with risk of pancreatic cancer after Bonferroni correction (*P*<1×10^−5^): *ABO, HNF1A, OR13C4,* and *SHH.* Functional enrichment analysis using DAVID consistently found the G protein-coupled receptor signaling pathway (including both neuroactive ligand-receptor interaction and olfactory transduction pathways) to be the most significant pathway for pancreatic cancer risk in this study population.

**Conclusion:**

These novel findings provide new perspectives on genetic susceptibility to and molecular mechanisms of pancreatic cancer.

## Introduction

Pancreatic cancer is the fourth leading cause of cancer-related death in the United States, accounting for more than 37,660 deaths per year [1]. Because no effective screening test exists for pancreatic cancer, it is important to identify genetic factors that contribute to the development of this cancer. Recent genome-wide association studies (GWAS) and post-GWAS analyses have identified chromosome regions containing the *ABO*, *NR5A2,* and *CLPTM1L-TERT* genes [2], [3], as well as the *HNF1A* gene [4], as susceptibility loci for pancreatic cancer. However, single-marker association tests have limited power to identify genes that are genuinely associated with disease status but may not reach a stringent genome-wide significance threshold in GWAS. Thus, many important disease genes may still remain unidentified with this approach. Furthermore, cancer development typically involves dysfunction of multiple functionally related genes acting concordantly in a network or pathways [5]. Thus, pathway analysis of GWAS data, which jointly considers multiple variants in interacting genes and multiple genes in a biological pathway, as a complementary approach to single-marker association tests [6], may have the potential to reveal the polygenic basis of disease susceptibility. Pathway-based GWAS analyses have provided novel insights into the etiology of cancers, such as colon cancer [7], lung cancer [8], and melanoma [9], and other complex diseases, including schizophrenia [10], bipolar disorder [11], and rheumatoid arthritis [12]. A recent study analyzed the GWAS data focusing on 23 selected pathways or groups of genes and identified the pancreas development pathway genes as susceptibility factors for pancreatic cancer [13]. While this data supports the candidate pathway analysis as a useful approach in genetic association study, it is limited by the number of pathways/genes examined, suggesting that a more comprehensive agnostic analysis of all known pathways may have the potential to uncover novel genes that were previously not considered in pancreatic cancer.

Gene set ridge regression in association studies (GRASS) is one of the newly developed pathway-based approaches [7]. In GRASS, principal components analysis (PCA) is used to capture the genetic variation within a gene to reduce the dimensionality of the single-nucleotide polymorphism (SNP) data and regularized logistic regression is performed to assess the association of pathways with disease. In this study, we first used GRASS on GWAS data to assess the association of pathways with pancreatic cancer. Then, we applied the logistic kernel machine (LKM) method to screen the major contributing genes to each pathway [14]. Finally, we conducted functional enrichment analysis of the most significant genes using the Database for Annotation, Visualization, and Integrated Discovery (DAVID) method [15], [16]. In this study, the first comprehensive analysis of GWAS data in pancreatic cancer using an agnostic approach, we have identified novel pathways and genes that are significantly associated with the disease risk. These findings may open new avenues of research on the molecular mechanism and etiology of pancreatic cancer.

**Table 1:** Pathways significantly associated with pancreatic cancer risk.

KEGG code	Pathway description	No. of genes	No. of SNPs/No.of eigenSNPs^a^	*P value* b	Major contributing genes^c^
hsa04080	Neuroactive ligand-receptor interaction	263	6116/1374	0.00002	CCKBR CHRM5 EDNRA LPAR1 NMUR1 P2RX4 SSTR3 F2RL3 OPRK1 GZMA S1PR2 SSTR2 CHRNB3 SCTR THRB CALCRL DRD4 VIPR1
hsa04664	Fc epsilon RI signaling pathway	77	1714/405	0.00012	RAF1 AKT3 RAC2 NRAS MAPK1 PLA2G2A FCER1G HRAS
hsa04730	Long-term depression	68	2649/540	0.00006	RAF1 ITPR2 NRAS MAPK1 GNAS GUCY1A2 PLA2G2A HRAS
hsa04950	Maturity onset diabetesof the young	24	299/91	0.00006	HNF1A HNF4G NR5A2 PDX1 HNF1B NEUROG3
hsa04270	Vascular smooth muscle contraction	113	3791/806	0.00024	ITPR2 RAF1 EDNRA ADCY9 KCNMA1 KCNMB2 GUCY1A2 GNAS PLA2G2A CALCRL MAPK1 PLA2G5 ADCY3 MYLK3
hsa04740	Olfactory transduction	353	4084/1122	0.0001	OR13C4 OR13C3 OR1L4 OR1L6 OR9G1 OR2G3 OR2G2 OR1L1 OR1L3 OR13C8 OR13C5 OR5M11 OR8B8 OR1N1 OR10P1 OR13C2 OR9G4 OR2H1 OR1J4 OR1J2 OR13C9 OR1N2 OR1L8 OR5M10 OR1C1 OR51G1 OR8U8 CNGA4 OR4A16 OR52W1 OR13A1 OR10H4 OR10C1 OR1M1 OR13F1 OR52B2 OR5M1 OR51B4 OR51A2 OR14A16 OR51F2 OR10G3 OR5L1 OR51A4 OR51G2

Note: All 6 pathways were significant after Bonferroni correction (*P*<2.50×10^−4^).

a “No. of SNPs” refers to the actual number of SNPs; “No. of eigenSNPs” refers to the number of uncorrelated linear combinations of SNPs used in GRASS analysis.

b *P* value obtained from GRASS analysis based on 50,000 permutations.

c Genes associated with pancreatic cancer with *P*<0.001, identified using the logistic kernel machine (LKM) method.

## Methods

### Study Population and Data Source

The study population included a total of 7,019 individuals: 1,871 cases and 2,026 from PanScan1 including 12 nested case–control studies and one hospital-based case control study and 1,528 cases and 1,594 controls from PanScan2 including 6 case–control studies on pancreatic cancer [2], [3]. Cases were defined as primary adenocarcinoma of the exocrine pancreas. Controls, which were free of pancreatic cancer at the time of recruitment, were matched to cases according to birth year, sex, and self-reported race/ethnicity. GWAS had been performed at the National Cancer Institute’s Core Genotyping Facility using the HumanHap550, HumanHap550-Duo, and Human 610-Quad arrays (all from Illumina, San Diego, CA) [2], [3]. The original GWAS data were downloaded from the Database of Genotypes and Phenotypes (dbGaP) [17]. On the basis of International HapMap Project genotype data (phase 3 release #3, NCBI build 36, dbSNP b126, 2010-05-28) for three populations (CEU, JPT/CHB, and YRI) [18] and minor allele frequency (MAF) >5%, we selected 10,155 SNPs with *r* ^2^<0.004 to use in population structure analysis [19]. A total of 6,508 individuals (3,141 cases and 3,367 controls) with European ancestry (i.e., 0.75–1 similarity to CEU) were selected from the starting study population of 7,019 individuals in the current pathway analysis.

**Table 2:** GRASS analysis of significant pathways in PanScan1 and PanScan2 dataset.

Pathway code	Pathway description	Combined	Panscan1	Panscan2	Stouffer's meta-p	Random1	Random2	Stouffer's meta-p	
hsa04080	Neuroactive ligand-receptor interaction	0.00002	0.00006	0.002	<1.0×10^−5^	0.0256	0.279	0.0365	
hsa04664	Fc epsilon RI signaling pathway	0.00012	0.11	0.18	0.065	0.0624	0.0076	0.0025	
hsa04730	Long-term depression	0.00006	0.1232	0.14	0.057	0.5422	0.0418	0.1254	
hsa04950	Maturity onset diabetes of the young	0.00006	0.00006	0.91	0.038	0.0856	0.0676	0.0215	
hsa04270	Vascular smooth muscle contraction	0.00024	0.10	0.10	0.035	0.357	0.0352	0.0620	
hsa04740	Olfactory transduction	0.0001	0.0015	0.002	1.0×10^−5^	0.0036	0.0816	0.0020	

### Quality Control

The original GWAS data passed quality control procedures before posted on dbGaP. We pruned the genotype data by further excluding 13,822 SNPs with call rate <98%, 45,653 SNPs with MAF <5%, and 38,857 SNPs deviating from Hardy–Weinberg equilibrium (*P*<0.001), as well as SNPs in the gene desert regions, resulting in 82,881 SNPs in the final analysis from a starting number of 468,111 SNPs.

**Figure 1:**
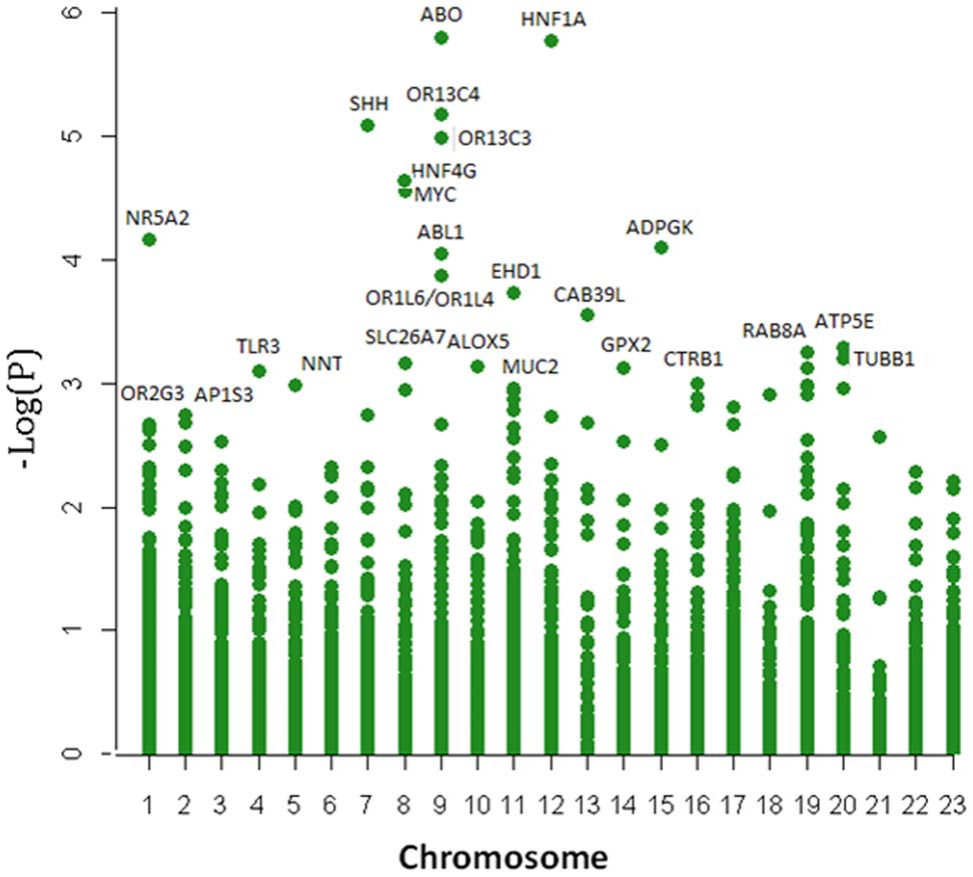
Genes significantly associated with pancreatic cancer (*P*<0.01 in LKM) in all 197 pathways analyzed in this study.

In order to evaluate the impact of population structure, we made the quantile-quantile (Q-Q) plot and calculated the inflation factor (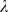) in individuals with European ancestry only. The inflation factor was calculated according to method by de Bakker et al. [20], adjusted for a sample size of 1,000 cases and 1,000 controls using the formula: 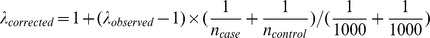
Where, 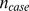 and 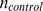 are actual number used to calculate 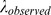; 1,000 is the sample size to be corrected. Q-Q plot shows little inflation of test statistics compared with expected distribution (λ = 1.03), excluding the possibility of potential population structure between cases and controls.

**Figure 2:**
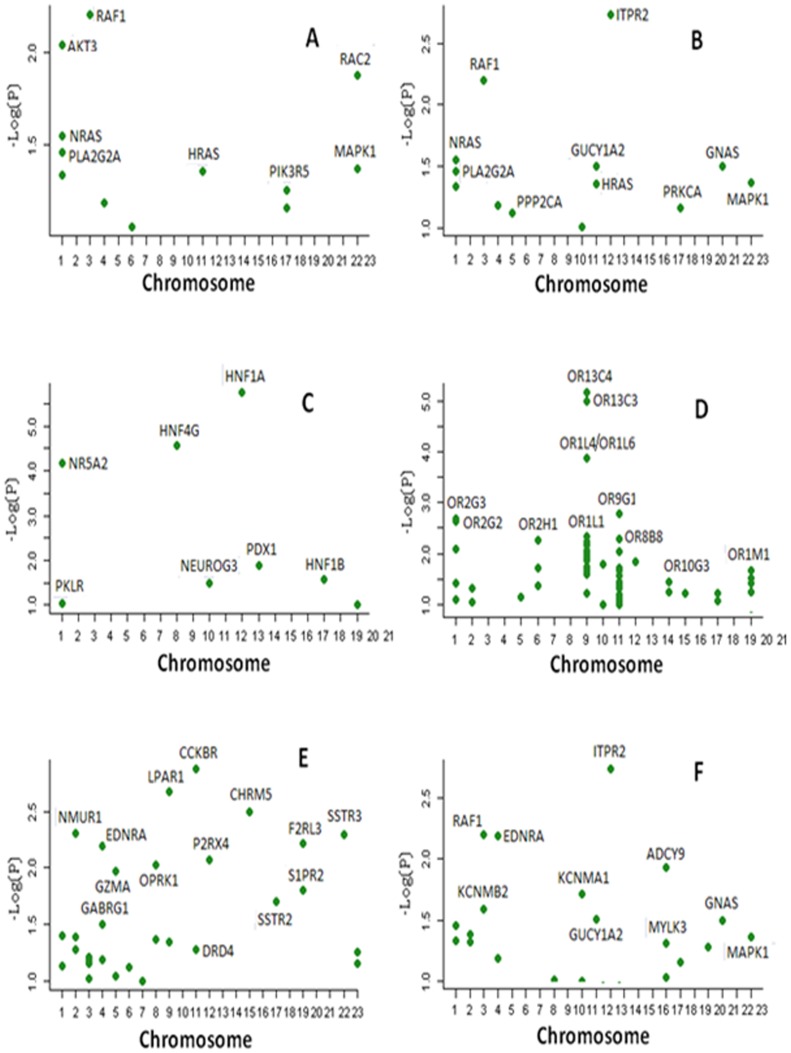
Genes significantly associated with pancreatic cancer in (A) the Fc epsilon RI signaling pathway; (B) long-term depression; (C) MODY; (D) olfactory transduction; (E) neuroactive ligand-receptor interaction pathway; and (F) vascular smooth muscle contraction pathways. Genes with –log10(*P*) <1 were not included in the plots. For clarity, not all genes are labeled. For details, see Tables S1 and S3.

**Table 3:** Genes associated with pancreatic cancer risk at *P* values <0.0001 in LKM analysis.

Gene	*P* value^a^	Full name	No. of SNPs/No. of eigenSNPs^b^	KEGG code
*ABO*	1.56×10^−6^	ABO blood group (transferase A, alpha 1-3-N-acetylgalactosaminyltransferase; transferase B, alpha 1-3-galactosyltransferase)	23/5	hsa00601
*HNF1A*	1.69×10^−6^	HNF1 homeobox A	16/4	hsa04950
*OR13C4*	6.63×10^−6^	olfactory receptor, family 13, subfamily C, member 4	13/4	hsa04740
*SHH*	8.18×10^−6^	sonic hedgehog	14/4	hsa05217 hsa05200 hsa04340
*OR13C3*	1.03×10^−5^	olfactory receptor, family 13, subfamily C, member 3	14/4	hsa04740
*HNF4G*	2.62×10^−5^	hepatocyte nuclear factor 4, gamma	14/5	hsa04950
*MYC*	2.73×10^−5^	v-myc myelocytomatosis viral oncogene homolog (avian)	4/1	hsa05222 hsa05221 hsa05220 hsa05219 hsa05216 hsa05213 hsa05210 hsa05200 hsa04630 hsa04350 hsa04310 hsa04110 hsa04012 hsa04010
*NR5A2*	6.76×10^−5^	nuclear receptor subfamily 5, group A, member 2	55/12	hsa04950
*ADPGK*	7.92×10^−5^	ADP-dependent glucokinase	8/3	hsa00010
*ABL1*	8.95×10^−5^	c-abl oncogene 1, non-receptor tyrosine kinase	30/7	hsa05416 hsa05220 hsa05200 hsa05131 hsa05130 hsa04722 hsa04360 hsa04110 hsa04012

Note: The first four genes were significant after Bonferroni correction (*P*<9.75×10^–6^).

a *P* value obtained from LKM analysis.

b “No. of SNPs” refers to the actual number of SNPs; “No. of eigenSNPs” refer to the number of uncorrelated linear combinations of SNPs used in GRASS analysis.

### Pathways and Genes

A total of 214 human biological pathways are listed in Kyoto Encyclopedia of Genes and Genomes (KEGG) [21]. After excluding pathways with <10 or >500 genes, we analyzed 197 pathways using the GRASS approach [22]. We identified 19,058 Reference Sequence (RefSeq) genes in the GWAS data from the human genome 18 (hg18) database using the University of California Santa Cruz (UCSC) Table Browser data retrieval tool [23]. We tested 5,127 genes in the 197 pathways for association with pancreatic cancer. For each gene region, we included SNPs within 20 kb upstream or downstream of the gene in this study.

**Figure 3:**
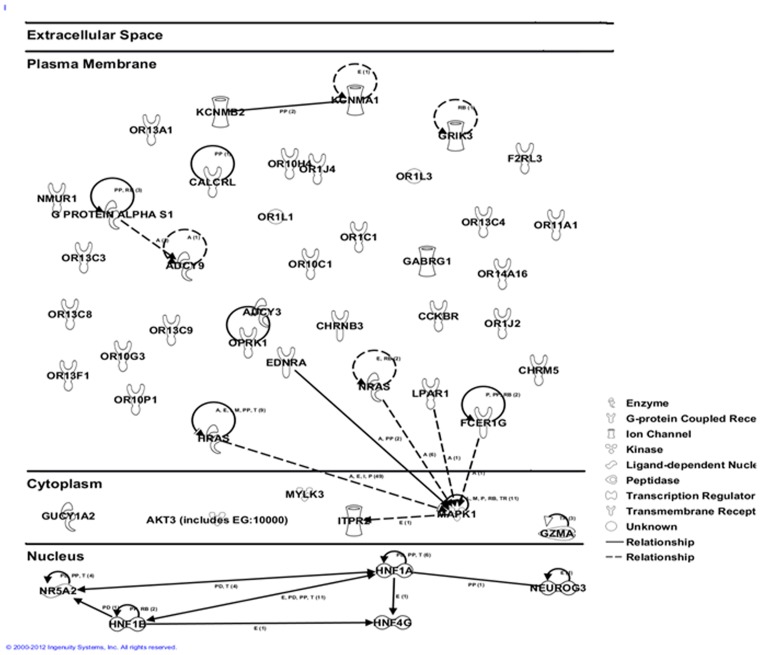
Ingenuity biologic systems map of the top 81 genes (*P*<0.05 in LKM) of the six pathways that are significantly associated with risk of pancreatic cancer. The solid line and dashed line, respectively, shows the direct and indirect interactions between genes.

**Table 4:** Functional enrichment analysis of top genes associated with pancreatic cancer (*P*<0.01 from KLM)^a^.

Cluster (Enrichment score^b^)	Biological process	Database code	No. of genes	*P* value^c^
1 (6.61)	GPCR, rhodopsin-like superfamily	IPR017452	26	1.61×10^−11^
	7TM GPCR, rhodopsin-like	IPR000276	26	1.66×10^−11^
	Olfactory receptor	IPR000725	18	5.11×10^−9^
	Sensory perception of smell	GO:0007608	18	1.30×10^−7^
	G-protein coupled receptor protein signaling pathway	GO:0007186	29	1.53×10^−7^
	Sensory perception of chemical stimulus	GO:0007606	18	5.60×10^−7^
	Cell surface receptor linked signal transduction	GO:0007166	36	2.58×10^−6^
	Sensory perception	GO:0007600	22	4.01×10^−6^
	Cognition	GO:0050890	23	6.91×10^−6^
	Neurological system process	GO:0050877	25	6.71×10^−5^
	Olfactory transduction	hsa04740	18	2.38×10^−3^
2 (1.97)	Homeostatic process	GO:0042592	18	1.97×10^−4^
	Chemical homeostasis	GO:0048878	14	4.03×10^−4^
	Cellular cation homeostasis	GO:0030003	9	1.45×10^−3^
	Activation of phospholipase C activity	GO:0007202	5	2.25×10^−3^
	Positive regulation of phospholipase C activity	GO:0010863	5	2.25×10^−3^
	Positive regulation of phospholipase activity	GO:0010518	5	2.80×10^−3^
	Cation homeostasis	GO:0055080	9	3.04×10^−3^
	Regulation of phospholipase activity	GO:0010517	5	3.11×10^−3^
	Positive regulation of lipase activity	GO:0060193	5	3.79×10^−3^
	Second-messenger-mediated signaling	GO:0019932	8	3.91×10^−3^
	Cellular ion homeostasis	GO:0006873	10	4.59×10^−3^
	Cellular chemical homeostasis	GO:0055082	10	5.09×10^−3^
	Cellular homeostasis	GO:0019725	11	6.33×10^−3^
	Regulation of lipase activity	GO:0060191	5	6.42×10^−3^
	Ion homeostasis	GO:0050801	10	8.10×10^−3^
	Activation of phospholipase C activity by G-protein coupled receptor protein signaling pathway coupled to IP3 second messenger	GO:0007200	4	9.77×10^−3^

a DAVID analysis was based on GO, InterPro, and KEGG database.

b The group enrichment score is the geometric mean (in -log scale) of member gene sets’ *P* values in a corresponding annotation cluster.

c The *P* value is based on a modified Fisher’s exact test in the DAVID system, referring to one-tail Fisher’s exact probability value used for gene-enrichment analysis.

### Statistical Methods

We used GRASS to test the association of each pathway with pancreatic cancer. Genotype data were coded in an additive model using PLINK version 1.07 [24] with 0 for homozygote common allele, 1 for heterozygote, and 2 for homozygote mutant allele. The GRASS tests the null hypothesis that none of the SNPs in a given pathway was associated with the disease [6]. To avoid undue influence of varying gene and pathway sizes, the GRASS uses normalized gene-level statistics and sample (subject) permutations. The details of GRASS have been previously described [7]. Briefly, the method consists of three steps. First, PCA is used to summarize SNPs in each gene as uncorrelated (orthogonal) linear combinations of the original SNPs, called eigenSNPs, accounting for ≥95% of the genetic variation. The number of resulting eigenSNPs is usually much smaller than that of the original genotyped SNPs and serves as predictors in the regularized logistic regression model. A penalized likelihood function is used to estimate the regression coefficients of the eigenSNPs. Second, a standardized gene-level statistic is calculated according to the regression coefficients of the eigenSNPs. The statistic, analogous to z-statistic, is defined as
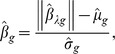
where



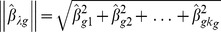



is the square root of summation of squared regression coefficient for each eigenSNP estimated under the optimal tuning parameter 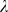; 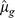 and 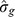, estimated from permutations, are mean and standard deviation of 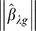
under the null hypothesis that gene 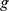 is not associated with the disease. Thus, each gene, regardless of its size, contributes equally to the gene set association statistic, as described below. The third step involves calculating gene set (pathway) association statistic (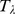) and p value. 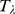 is the square root of summation of squared standardized gene-level statistics; *P* value is estimated using 
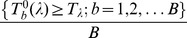
, where 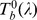 is computed from the permutated data and B is the number of permutations. Because of the large number of genes and pathways analyzed, we applied the Bonferroni correction to adjust for multiple comparisons. The significance threshold was *P*<0.00025 (0.05/197). Due to the intensive computation entailed by GRASS, we adopted a two-stage permutation test procedure, similar to that implemented in PLINK [24]: we first conducted 5,000 permutations to each gene set in this study, and for those gene sets with p-value less than 0.00025, we increased the number of permutations to 50,000.

We applied the LKM test to assess the association of each gene with pancreatic cancer as previously described [14]. Briefly, this method comprises two steps: forming SNP sets for each gene and testing the association of SNP sets with disease status. The gene database, gene region definition, and genotype coding used here were the same as those in GRASS. The LKM model integrates a regular logistic model with a semi-definite kernel function (a linear kernel was used here) that is specifically designed for genetic data. The variance-components score test of Zhang and Lin [25] was used to test gene–disease association. In this analysis, we tested the associations of 5,127 genes (in the 197 pathways) with pancreatic cancer after adjusting for age (in 10-year groups), sex, study (categorical), and five principal components (quantitative) capturing population structure obtained from a PCA analysis using EIGENSTRAT [26]. P values from the KLM analysis were adjusted for multiple comparisons using the Bonferroni correction. The significance threshold was *P*<9.75×10^−6^ (0.05/5,127).

Finally, as a complementary approach to the GRASS pathway analysis, we investigated the functional enrichment of the most significant genes in gene-based association tests (*P*≤0.01 in LKM) using the web-accessible bioinformatics tool DAVID [15], [16]. The DAVID consists of an integrated biological knowledgebase and analytic tools aimed at systematically extracting biological meaning and over-represented biological functions from large gene or protein lists based on the hypergeometric (Fisher’s exact) test. We used the KEGG, GO and InterPro [27] databases to define the gene sets. In addition, DAVID groups functionally similar gene sets into clusters to reduce the redundant nature of gene functional annotation systems, e.g., the hierarchically organized GO.

As a replication effort, we analyzed the data from PanScan1 (1,796 cases and 1,880 controls) and PanScan2 (1,345 cases and 1,487 controls) separately. We also randomly split the entire dataset into two groups and conducted separate analysis in each group. We performed meta-analysis of the *P* values from individual cohort/group using the Stouffer’s z-score method, which has been demonstrated to be efficient in meta-analysis of GWAS [28]. The test statistic for combining p-values from two individual cohorts for a given pathway is computed as 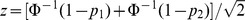, where 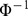 is inverse of the standard normal cumulative function. The overall meta-analysis *P* value is calculated as 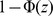. Finally, we applied the GRASS method to test the two largest significant pathways (as detailed in Results) using the Wellcome Trust Case Control Consortium (WTCCC) GWAS data [29] to empirically evaluate the impact of pathway size and demonstrate the specificity of our results.

## Results

### Pathways Associated with Pancreatic Cancer

We analyzed 197 pathways with 5,127 genes using the GRASS approach and found that six pathways were significantly associated with pancreatic cancer after the Bonferroni correction (*P*<2.5×10^−4^) (Table 1). Three pathways were significant at *P* values <0.0001: neuroactive ligand-receptor interaction, long-term depression, and maturity onset diabetes of the young (MODY) pathways. Three pathways had a less significant *P* value of ≥0.0001 but <0.00025: the olfactory transduction, Fc epsilon RI signaling, and the vascular smooth muscle contraction pathways. In addition to the above six pathways, the glycerophospholipid metabolism, pancreatic secretion and vascular endothelial growth factor (VEGF) signaling pathways were associated with pancreatic cancer at *P = *0.0004 (Table S1, available online).

### Pathway Replication Results

Two of the six significant pathways, i.e. the olfactory transduction pathway and the neuroactive ligand-receptor interaction pathway showed consistent small *P* values across the PanScan1 and PanScan2 cohorts, though not both *P* values were significant after multiple testing corrections likely due to much smaller sample size in each individual cohort and the resulting lower statistical power (Table 2). The meta-analysis *P* values for these two pathways (1×10^−5^ and <1×10^−5^, respectively) were significant after the Bonferroni correction. The MODY pathway remained significant in PanScan1 (*P* = 0.00006) but not in PanScan2 (*P* = 0.91), and the meta-analysis *P* value was 0.0589. All three remaining significant pathways in the combined GRASS analysis had *P* values >0.1 in the PanScan1 and PanScan2 cohort (Table 2). When we randomly split the dataset into two groups, all six pathways had a *P* value <0.05 in one of the two groups but not in both; and none of the meta-analysis *P* values was significant after adjusting for multiple comparisons (Table S2). To investigate if the significance of the olfactory transduction pathway (353 genes and 1,122 eigenSNPs) and the neuroactive ligand receptor interaction pathway (263 genes and 1,374 eigenSNPs) was simply due to their large size, we tested these pathways by applying the GRASS to the WTCCC GWAS data and obtained a *P* value of 0.5652 and 0.2332 for bipolar disorder and 0.246 and 0.0062 for Crohn’s disease, respectively, (each disease had 2,000 cases and 3,000 controls). These results, along with the consistent small *P* values across PanScan1 and PanScan2, indicate that the significant *P* value of these two pathways in the GRASS analysis is unlikely due to the pathway size. In addition, to investigate if the pathway results were mainly driven by GWAS top hits reported in PanScan1 and PanScan 2, we removed the gene *NR5A2* from the MODY pathway and re-performed GRASS analysis with 50,000 permutations. The P value for the combined dataset, PanScan1 and PanScan2 subset was 0.4×10^−4^, 0.6×10^−4^, and 0.88, respectively. The respective *P* values were 0.6×10^−4^, 0.6×10^−4^ and 0.91 from the analysis including the *NR5A2* gene, suggesting that our pathway analysis unraveled signals independent from those by single-SNP analysis. Note that other GWAS top hits such as *ABO* and *TERT1* were not included in any of the 197 pathways.

### Major Contributing Genes to Pathways

Applying the LKM method, we identified 365 genes with nominal significance (*P*<0.05) and 118 genes with *P*<0.01 for the 197 pathways (Table S3, available online). The major contributing genes to each of the six significant pathways identified by GRASS are listed in Table 1. The major genes contributing to the 197 pathways and to the six significant pathways are presented in Figure 1 and 2, respectively. After we adjusted for multiple comparisons, four genes remained significant (*P*<9.75×10^−6^): the *ABO*, *HNF1A*, *OR13C4*, and *SHH* genes (Table 3). In addition to these four genes, *ABL1*, *MYC, HNF4G*, *NR5A2* (a GWAS top hit) and *ADPGK* had *P* values <0.0001.

### Functional Enrichment Analysis of Significant Genes

Finally, we conducted functional enrichment analysis, using DAVID, on the set of 118 genes with *P*<0.01 from LKM analysis. Forty-four clusters were identified on the basis of the KEGG, GO and InterPro categories. The clusters of genes with *P*<0.01 from DAVID are listed in Table 4 (See Table S4 for detailed list of genes in each cluster). The superfamily of rhodopsin-like G protein-coupled receptors (GPCRs), perception of smell and olfactory transduction was the most significant group of genes on the basis of the InterPro (*P* = 1.61×10^–13^), GO (*P* = 1.30×10^–7^) and KEGG (2.38×10^−3^) databases, respectively, echoing our findings from GRASS. Genes maintaining the homeostasis process were also over-represented in pancreatic cancer (Table 4). The biologic relationship map for the top 81 genes (*P*<0.05 in LKM) of the six significant pathways is shown in Figure 3, which was created with Ingenuity Pathway and Analysis software [30].

## Discussion

In this GWAS pathway analysis, we identified two novel pathways, i.e. the neuroactive ligand receptor interaction and olfactory transduction pathways that are significantly associated with pancreatic cancer risk after adjusting for multiple comparisons and in replication testing. These findings were also supported by functional enrichment analysis. We also identified four genes that are significantly associated with pancreatic cancer risk, including three previously implicated genes *ABO*, *HNF1A*, and *SHH* [2]–[4] as well as a novel gene *OR13C4*. These findings provide new provocative insights into the polygenic basis of pancreatic cancer susceptibility and etiology.

The GPCR protein superfamily of transmembrane receptors accounts for ∼4% of the whole human genome and >50% of modern therapeutic targets [31]. Genes of the neuroactive ligand-receptor interaction and olfactory transduction pathways are major components of the GPCRs (Table S4). The neuroactive ligand-receptor interaction pathway remained significant after adjusting for multiple testing in PanScan1 (*P* = 0.0006) but not in PanScan2 (*P* = 0.002). However, the P value from meta-analysis was highly significant (*P*<1×10^−5^). The contributing genes to this pathway, e.g. *CCKBR, CHRM5, EDNRA, LPAR1*, *SSTR2/3*, and *SCTR*, have diverse functions in regulating the endocrine and exocrine functions of the pancreas, which are highly relevant to pancreatic cancer [32], [33], [34], [35].

Humans have >700 olfactory receptor (OR) genes (of which ≥50% are functional) [36]. Genetic variants of the OR genes and dysfunctions of OR signaling have previously been associated with schizophrenia [37], fetal hemoglobin in sickle-cell anemia [38], and proliferation of prostate cancer cells [39]. Although the links between olfactory transduction and pancreatic cancer remain to be elucidated, a previous sequencing analysis of human pancreatic tumors did find many somatic mutations of the OR genes, including seven genes identified in the current analysis: *OR13C3*, *OR13C5*, *OR10P1*, *OR1J2*, *OR4A16*, *OR51F2*, and *OR5D13* [40]. Expression of at least two OR genes has been reported in human pancreas tissues [41]. The top two contributing genes to the olfactory transduction pathway, *OR13C4* and *OR13C3*, ranked as the third and fifth most significant genes among the 5,127 genes analyzed in this study. In the replication study, the olfactory transduction pathway remained as one of the top pathways with consistent small *P* values in PanScan1 and PanScan2 cohort with a significant meta-analysis *P* value after adjusting for multiple testing. On the other hand, we did not find any association of this pathway with bipolar or Crohn’s disease in the WTCCC GWAS data analysis. All these data suggest that the association of olfactory transduction pathway and pancreatic cancer are unlikely to be due to chance. Further replication of this association in other dataset and functional studies of the biological and molecular links between olfactory transduction signaling and pancreatic cancer are warranted. GPCRs are the first gate through which outside signals are transmitted into the cell. High activity of GPCRs may contribute to transduction of outside detrimental signals, such as insulin, glucose, or carcinogens, into a cell and induce a cascade of responses related to carcinogenesis.

In addition to these two pathways, four additional pathways also passed the Bonferroni correction for multiple comparisons, i.e. the MODY, Fc epsilon RI, long-term depression and vascular smooth muscle contraction pathways. However, the MODY pathway was highly significant in PanScan1 but was not significant in PanScan2. The diminished or weaker gene-risk association in PanScan2 has previously been observed for other genes [3], [4]. This difference may be related to the fact that PanScan1 was pooled from 12 cohort studies and one case control study while PanScan2 was drawn from eight case control studies. Because of the rapid fatality of pancreatic cancer, case control study may subject to a survival bias if the testing genes are associated with survival. Although meta-analysis did not show a significant *P* value, this pathway has been identified as the most significant pathway in association with pancreatic cancer in a separate analysis of the PanScan data using two different statistical methods [13]. The MODY genes are an important part of the transcriptional network that regulates pancreas development and differentiation in early life and maintains pancreatic homeostasis in adulthood [42], [43], [44]. Three MODY genes (*HNF1A*, *HNF4G*, and *NR5A2*) were among the top 10 genes with *P* values <0.0001 in LKM analysis. Notably, another two of the top 10 genes, *SHH* and *MYC*, are also known to play an essential role in pancreas development [45]. Genes involved in organ development and differentiation may contribute to the ability of tumor cells to proliferate and survive, as well as alter cell plasticity, thus reprogramming cells to a state that can give rise to a tumor. MODY genes may also contribute to pancreatic cancer by modifying the risk of diabetes [46] and obesity [47], [48], or by regulating epithelial cell growth and differentiation, lipid metabolism [49], protein fucosylation [50], and inflammation [51].

Fc epsilon RI is a high affinity receptor for IgE, and mast cell activation mediated by Fc epsilon RI is a key event in the allergic inflammatory response. Increasing evidence indicates that inflammation around tumor, including infiltration by mast cells, facilities tumor growth and angiogenesis in pancreatic cancer [52], [53]. However, this pathway along with the long-term depression and vascular smooth muscle contraction pathways did not have consistent results in the replication studies. Thus, these data need to be treated with caution.

Compared to findings from the previously reported candidate pathway/gene analysis [13], our findings on pathways that were included in both studies were quite consistent, i.e. a positive finding on the pancreas development (aka MODY) pathway/genes and the negative findings on the DNA repair, apoptosis, insulin signaling, wnt, notch and hedgehog pathways/genes.

This is by far the largest study in pancreatic cancer with the most comprehensive analysis of all biological pathways identified from KEGG using an agnostic approach. Using PCA in GRASS greatly reduced the dimensionality of the GWAS data and increased the probability of singling out useful information. Using the LKM method overcame the influences of positive and negative effects of SNPs and enabled us to identify new genes in addition to replicating the gene regions discovered by previous marginal-association studies [54]. By performing the GRASS analysis in two independent cohorts, we have shown consistent findings on some of the significant pathways. Further replication of these findings in future additional pancreatic cancer GWAS data is warranted. Overall, the pathway analysis approach with intensive control for false positive findings has a great potential to uncover gene traits that are associated with disease without a priori. Correct use of this tool may open up new avenues of research on the molecular mechanisms of pancreatic cancer and potential targets for the prevention and treatment of this disease.

## Supporting Information

Table S1 List of 197 biological pathways analyzed in this study.
(XLS)

Table S2 Results of GRASS analysis in subgroups and in WTCCC GWAS dataset.
(XLS)

Table S3 List of 5,127 genes analyzed in this study.
(XLS)

Table S4 List of genes in each cluster identified in DAVID analysis.
(XLS)
